# Amino Acid Patterns around Disulfide Bonds

**DOI:** 10.3390/ijms11114673

**Published:** 2010-11-18

**Authors:** José R. F. Marques, Rute R. da Fonseca, Brett Drury, André Melo

**Affiliations:** 1 REQUIMTE/Departamento de Química e Bioquímica, Faculdade de Ciências da Universidade do Porto, Rua do Campo Alegre, 687, 4169-007 Porto, Portugal; E-Mail: zerui.marques@fc.up.pt; 2 CIMAR/CIIMAR, Centro Interdisciplinar de Investigação Marinha e Ambiental, Universidade do Porto, Rua dos Bragas, 177, 4050-123 Porto, Portugal; E-Mail: rute.r.da.fonseca@gmail.com; 3 LIAAD-INESC, Rua de Ceuta, 118, 6°, 4050-190 Porto, Portugal; E-Mail: brett.drury@gmail.com

**Keywords:** disulfide bond, neighborhood, classification, frequency, diversity

## Abstract

Disulfide bonds provide an inexhaustible source of information on molecular evolution and biological specificity. In this work, we described the amino acid composition around disulfide bonds in a set of disulfide-rich proteins using appropriate descriptors, based on *ANOVA* (for all twenty natural amino acids or classes of amino acids clustered according to their chemical similarities) and Scheffé (for the disulfide-rich proteins superfamilies) statistics. We found that weakly hydrophilic and aromatic amino acids are quite abundant in the regions around disulfide bonds, contrary to aliphatic and hydrophobic amino acids. The density distributions (as a function of the distance to the center of the disulfide bonds) for all defined entities presented an overall unimodal behavior: the densities are null at short distances, have maxima at intermediate distances and decrease for long distances. In the end, the amino acid environment around the disulfide bonds was found to be different for different superfamilies, allowing the clustering of proteins in a biologically relevant way, suggesting that this type of chemical information might be used as a tool to assess the relationship between very divergent sets of disulfide-rich proteins.

## 1. Introduction

Cysteine’s (*CYS*) ability to dimerize makes it unique among the twenty natural amino acids. A disulfide bond is formed between two oxidized *CYS* thiol groups. Disulfide bonds induce conformational restrictions on proteins strongly influencing their folding, stability and function [1–5].

Disulfide topology has been successfully used for protein clustering, where the disulfide structure was found to be well-conserved even for apparently non-related proteins [6–11]. The disulfide topology has been subsequently used to establish evolutionary relationships not detected by sequence similarity based methods. Disulfide three-dimensional structure and connectivity are highly conserved patterns in nature, and have become the basis of several protein classification analyses [12–19].

The stabilization of disulfide bonds has also been the focus of various studies. These include: (i) The analysis of the protein environment in the neighborhood of both bonded and free cysteines [20,21]; (ii) the geometrical requirements of a disulfide bond [21–23]; (iii) the influence of pH [14]; (iv) the role of redox mediators [23–25]; (v) the role of allosteric factors [26,27].

We have performed a systematic investigation on the amino acid composition around disulfide bonds of a set of disulfide-rich proteins selected according to their *SCOP* (Structural Classification of Proteins) classification [28–^30^]. Our goal was to assess whether or not the observed patterns can be used to group the proteins according to their biological characteristics, and therefore be used as a classification criteria for very divergent proteins. In our previous work [6], we demonstrated that the conformational patterns of disulfide bonds are sufficient to group proteins that share both functional and structural characteristics.

The protein set included twelve disulfide-rich protein superfamilies (according to the *SCOP* classification) that obeyed the following criteria: (i) contain a minimum of thirty disulfide bonds; (ii) have a minimum of five *PDB* structures available; (iii) have X-ray structures with a resolution higher than 2.5 Å and (iv) have only uncomplexed structures. The proteins belonged to the thioredoxin-like superfamily and eleven superfamilies containing small disulfide-rich proteins (*SDP*). The thioredoxin-like superfamily is very different from the other proteins in the set, namely because it: (i) presents a lower number of disulfide bonds per *PDB* structure; (ii) has an extensive hydrophobic core, completely absent in the small disulfide-rich proteins; (iii) is constituted by disulfide oxidoreductase enzymes; (iv) has a very structured secondary structure, compared to the few secondary structure elements characteristic of the small disulfide-rich proteins; (v) displays absence of disulfide cooperative effects (in small disulfide-rich proteins the disulfide and the buried side-chain influence the dynamics of the folded protein through stabilization effects resulting from the spatial proximity of two or more disulfide bonds) [12].

Other authors have analyzed the importance of the amino acid environment around disulfide bonds for the stabilization of 3D-structures in proteins [20,21] but to date no studies have attempted to use this type of chemical information to aggregate a set of proteins into their respective superfamilies. This is the main purpose of the present work. Our approach involved the use of stratified statistics, which groups the members of a population (the various proteins) into relatively homogeneous and orthogonal subgroups (the described superfamilies) before sampling.

## 2. Materials and Methods

### 2.1. General

We used three different criteria to describe the amino acid composition in the proximity of disulfide bonds: (i) all twenty natural amino acids were considered as independent units; (ii) the same amino acids were grouped into classes according to their chemical properties, and these classes clustered into two classification groups (Table 1). Each entity (amino acid or class) was characterized both by a relative frequency and a diversity index. As a reference set we used a number of proteins selected from the *PDB* database by Xia and Xie [31]. The protein set under study is characterized in Table 2. A list of all the *PDB* structures analyzed is available in Table 1 of Supplementary Material. A most frequent motif, combining *SCOP* clustering and structural elements, was also identified.

The analysis of the amino acid composition around disulfide bonds and the classification of the amino acid were carried using our program *Disulph* (see Table 2 in Supplementary Material for details on *Disulph* functionalities). This program, written in FORTRAN, also calculates the relative frequency and the density of each entity in the neighboring region of a disulfide bond in twenty pre-determined spherical shells with thickness 0.5 Å. The neighboring region of a disulfide bond was defined as a sphere, with radius 10 Å, centered at the middle point of this bond, and excluding the cysteines involved in the bond (Table 3 in Supplementary Material). All the residues containing at least an atom in that region were considered for the statistical analysis. We calculated the conservation of the different entities over different superfamilies using the relative frequency of each entity in the neighboring region of all disulfide bonds. We performed: (i) a one-way *ANOVA* hypothesis test with a significance of 5% for each entity (residue or class); (ii) a Scheffé test, with the same significance, for each entity and pair of superfamilies.

### 2.2. Calculation of the Relative Frequencies for Each Entity

The relative frequency of entity *A*, in the neighborhood of disulfide *j*, present in superfamily *m*, is given by:

(1) $$\text{rel}\;\;\;\text{freq}{\left(A\right)}_{m,j}=(\text{freq}{\left(A\right)}_{m,j}-{\text{freq}}_{\mathrm{reference}}(A))/{\text{freq}}_{\text{reference}}(A)$$

where *freq_referece_*(*A*) is the frequency of the same entity in the reference set.

The relative frequency of entity *A*, for the superfamily *m*, that presents *nSS_m_* disulfide bonds, is given by:

(2) $$\text{rel}\;\;\text{freq}{\left(A\right)}_{m}=\sum_{j=1}^{nSS_{m}}\text{rel}\;\;\text{freq}{\left(A\right)}_{m,j}/nSS_{m}$$

Considering a set with *nSF* superfamilies, the relative frequency of the entity in the sample (*rel freq*(*A*)) can be calculated by:

(3) $$\text{rel}\;\;\text{freq}(A)=(1/nSF)\sum_{m-1}^{nSF}\sum_{j=1}^{nSS_{m}}\text{rel}\;\;\text{freq}{\left(A\right)}_{m,j}/nSS_{m}$$

### 2.3. ANOVA Test

Considering *nSS_total_* as the total number of disulfide bonds in the protein set under study, we can now calculate two auxiliary quantities, (i) the mean-square error between the superfamilies (*MS_betweenSF_*(*A*)) and (ii) the mean-square error within the superfamilies (*MS_withinSF_*(*A*)):

(4) $${\text{MS}}_{\text{betweenSF}}(A)=\sum_{m=1}^{nSF}nSS_{m}{\left(\text{rel}\;\;\text{freq}{\left(A\right)}_{m}-\text{rel}\;\;\text{freq}(A)\right)}^{2}/\left(nSF-1\right)$$

and

(5) $${\text{MS}}_{\text{withinSF}}(A)=\sum_{m=1}^{nSF}\sum_{j=1}^{nSS_{m}}{\left(\text{rel}\;\;\text{freq}{\left(A\right)}_{m,j}-\text{rel}\;\;\text{freq}{\left(A\right)}_{m}\right)}^{2}/\left(nSS_{\text{total}}-nSF\right)$$

The statistical parameter *F*, associated with the one-way *ANOVA* test carried out for entity *A*, is calculated as a quotient between the two mean-square error values:

(6) $$F={\text{MS}}_{\text{betweenSF}}(A)/{\text{MS}}_{\text{withinSF}}(A)$$

This parameter should be interpreted as:

- If *F* < Fcritical, the relative frequency of the considered entity should be equal for all the superfamilies (null hypothesis).
- If *F* > Fcritical, the mentioned frequency should be different for at least two superfamilies (alternative hypothesis).

In the present case, *F_critical_* = 1.8 and the null hypothesis never occurs.

Alternatively, the statistical parameter *F* can also be interpreted as a diversity index. The diversity of the associated entity over the sample increases when *F* increases. On the other hand, this diversity decreases over the sample when *F* decreases. The statistical parameter *F* is invariant with respect to any linear transformation. This means that, using this statistical index, diversity is a property intrinsically associated with the data sample and completely independent of the reference set considered.

### 2.4. Scheffé Test

Complementary to the one-way *ANOVA* statistics carried out for entity *A*, we performed the Scheffé test to compare the variability associated with two superfamilies *m* and *l*. The correspondent statistical parameter $F_{m,l}^{\text{Scheffe}}(A)$ is defined as:

(7) $$F_{m,l}^{\text{Scheffe}}(A)={\left(\text{rel}\;\;\text{freq}{\left(A\right)}_{m}-{\text{rel}\;\;\text{freq}(A)}_{l}\right)}^{2}/({\text{MS}}_{\text{withinSF}}(A)\times (1/nSS_{m}+1/nSS_{l})\times (nSF-1))$$

This parameter has the same invariance properties of the statistics parameter *F*, defined for a one-way *ANOVA* test, and should be interpreted in a similar way:

(iii) If $F_{m,l}^{\text{Scheffe}}(A)$ < Fcritical, the relative frequency of the considered entity should be equal for the superfamilies *m* and *l* (null hypothesis).
(iv) If $F_{m,l}^{\text{Scheffe}}(A)$ > Fcritical, the same frequency should differ for these two superfamilies (alternative hypothesis).

In the present case, *F_critical_* = 1.8 and the null hypothesis frequently occur. However, the presentation of these results would be difficult, because 27 entities were analyzed. Therefore, we would have to present 31 tables. So, in order to present the differences in the chemical environment around disulfide bonds, we developed new descriptors designated by Scheffé distances. A Scheffé distance $D_{m,l}^{\text{Scheffe}}$ compares the chemical environment around disulfide bonds between two superfamilies *m* and *l* for any classification group with *nE* entities:

(8) $$D_{m,l}^{\text{Scheffe}}=(1/nE)\times \sum_{A=1}^{nE}F_{m,l}^{\text{Scheffe}}(A)$$

### 2.5. Representing the Distances between Superfamilies

In order to represent distances ( $D_{m,l}^{\text{Scheffe}}$), inferred from the original 12-dimensional hyper-space, we adopted the intuitive form introduced by Xie *et al.* [32]. The coordinates of the original objects (the 12 superfamiles) are projected in the 3D Cartesian space by minimizing the square deviation cost function *SD*:

(9) $$SD=\sum_{m=1}^{nSF}\sum_{l=1}^{m-1}{\left(d_{l,m}-D_{m,l}^{\text{Scheffe}}\right)}^{2}$$

where *d_l,m_* is the distance between the projections the superfamilies *m* and *l* in the 3D Cartesian space. We used the Newton method to carry out the iterative minimization process.

### 2.6. Density of an Entity

The density of entity *A* within a spherical shell *i* of volume *V_i_* where *A* occurs *n*(*A*)*_i_* times for the all the disulfide bonds included in the sample can be calculated as

(10) $$d{\left(A\right)}_{i}=n{\left(A\right)}_{i}/V_{i}$$

### 2.7. Disulfide Bonds Propensity

The disulfide bonds propensity *Pr_m_*, for a superfamily *m* with *nPDB_m_* PDB structures, is calculated as,

(11) $${\text{Pr}}_{m}=(1/nPDB_{m})\sum_{k=1}^{nPDB_{m}}100\times nSS_{k}/nres_{k}$$

where *nSS_k_* and *nres_k_* are respectively the number of disulfide bonds and the number of natural amino acids in the PDB structure *k*.

## 3. Results and Discussion

### 3.1. Frequency and Density

**Figure 1.**
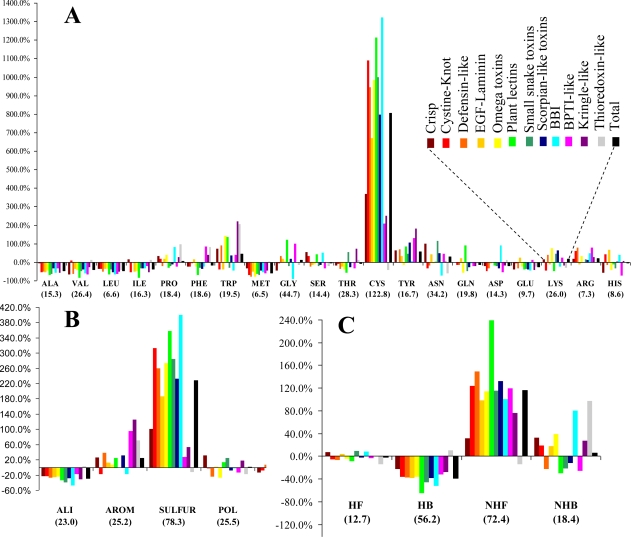
Relative frequencies around disulfide bonds of (**A**) the natural amino acids, (**B**) classes in classification group 1, and (**C**) classes in classification group 2. The black columns represent the relative frequencies for the sample. The other columns represent the relative frequencies for each superfamily. The values of the statistical parameter *F* associated with the one-way *ANOVA* test are presented in parenthesis.

The relative frequencies of the various entities and the corresponding values of the statistical parameter *F* are presented in Figure 1. Cysteines are by far the most abundant amino acid around disulfide bonds, placing the class *SULFUR* on top of the most abundant classes (even though methionine has the lowest relative frequency of all amino acids). Almost all these cysteines are disulfide bonded, preventing mis-pairing effects. This predominant abundance results from the *SDP* patterns, associated with the above mentioned disulfide cooperative effects. In the thioredoxin-like proteins, which present the lowest disulfide propensities, the cysteine is less abundant than in the reference set. Weakly hydrophilic and aromatic amino acids are abundant when close to disulfide bonds, particularly tyrosine and tryptophan. Aliphatic and hydrophobic amino acids exhibited the lowest relative abundance, particularly alanine, valine leucine and isoleucine. Positively charged amino acids (arginine and lysine) are very abundant in the neighborhood of disulfides, but since negatively charged groups disrupt these bonds glutamate and aspartate have a very low relative frequency. Accordingly, disulfides involving cysteines located at the *C*-terminal of a protein are rarely spotted.

The abundance, evaluated by a relative frequency, provided valuable information on the general trends observed in the sample. Although different protein sets and methodologies were used, our results are reasonably consistent to those obtained by Petersen *et al.* [21]. In fact, both studies are in agreement relatively to four of the five residues with highest abundance (cysteine, tryptophan, tyrosine and arginine). Aliphatic and hydrophobic amino acids exhibited the lowest relative abundance in both studies.

**Figure 2.**
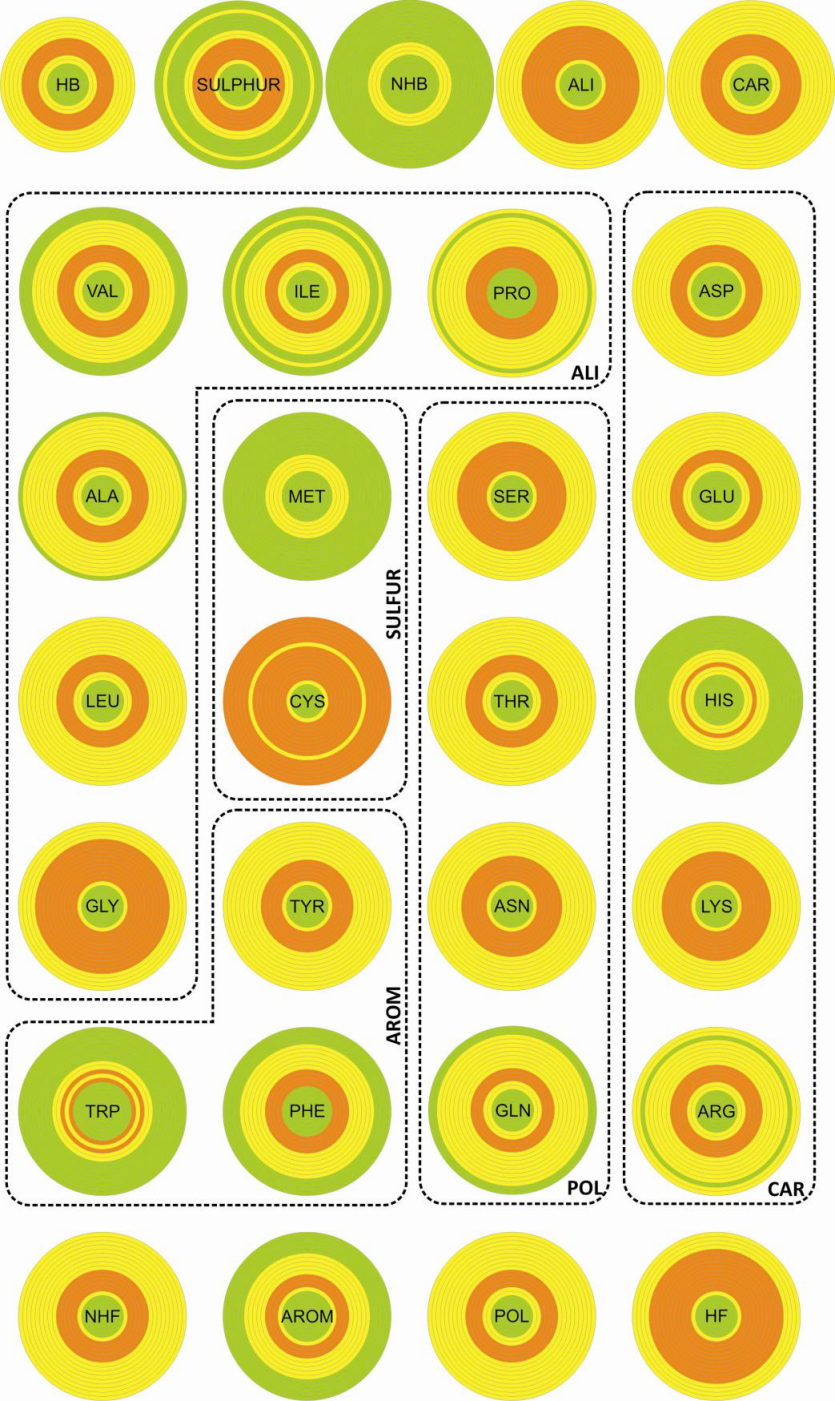
Densities for the twenty natural amino acids and the various classes in the different spherical shells. The following color notation is adopted: green means a density 50% smaller than a uniform density; yellow represents a density between 50% and 150% this density; and orange corresponds to 150% larger than the same reference.

The densities for the twenty natural amino acids and the different entities in the various spherical shells (Table 3 in Supplementary Material) are shown in Figure 2. The density distributions of the different entities as a function of the distance to the center of the disulfide bond display a common pattern: The densities are null at short distances, have maxima at intermediate distances and decrease for long distances.

Interestingly, we can see very different patterns for residues with similar relative frequencies. Among those that are on top of the frequency values (Table 4 in Supplementary Material), cysteine is the one showing an almost uniform distribution with high concentration practically everywhere from 2 to 10 Å distance from the disulfide bond. Tyrosine and tryptophan which have relative frequency values of around 50% show radically different distributions: Tyrosine is abundant in all shells and tryptophan is only significantly present at a distance of 3.5–6 Å from the disulfide bond.

### 3.2. Diversity

The entities (*CYS*, *SULFUR* and *NHF*) with highest relative abundance are associated with the largest diversity. However, the two quantities do not present any significant correlation.

**Figure 3.**
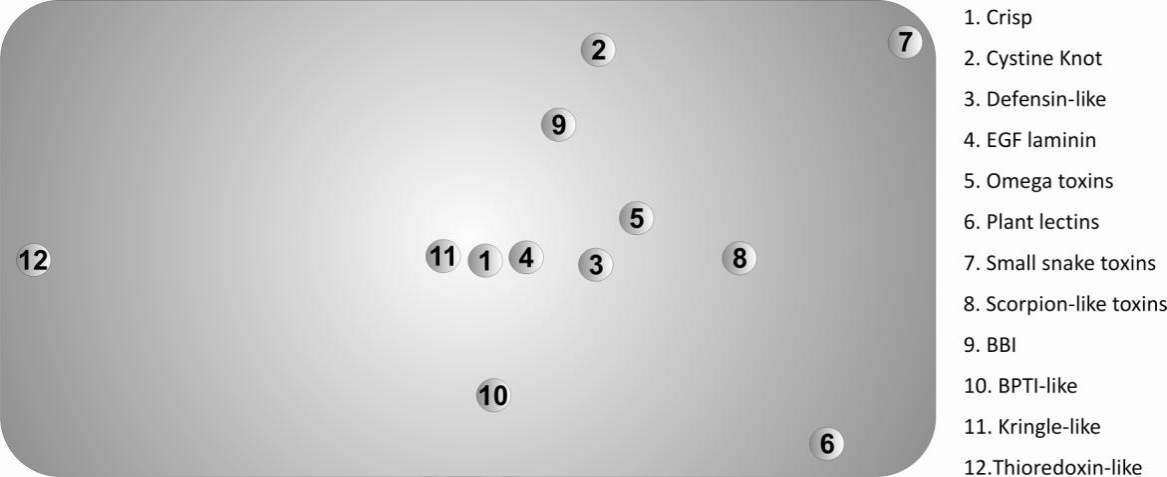
Projected 3D-Cartesian representation of the twelve superfamilies under study, inferred from the Scheffé distances calculated on the original 20-dimensional space of the natural amino acid.

The Scheffé distance matrices, obtained with the three classification criteria used in this work, were in reasonable agreement. In this context, we opted to represent only the projected 3D-Cartesian coordinates inferred from the 20-dimensional of natural amino acids in Figure 3.

These descriptors allowed us to find the superfamilies that present similar/dissimilar chemical environments around their disulfide bonds, providing useful information regarding evolutionary processes and further insight into the classification of disulfide-rich proteins. The main divergences, observed in Figure 3, can be explained by significant deviations from the most frequent motif identified in Table 2.

**Figure 4.**
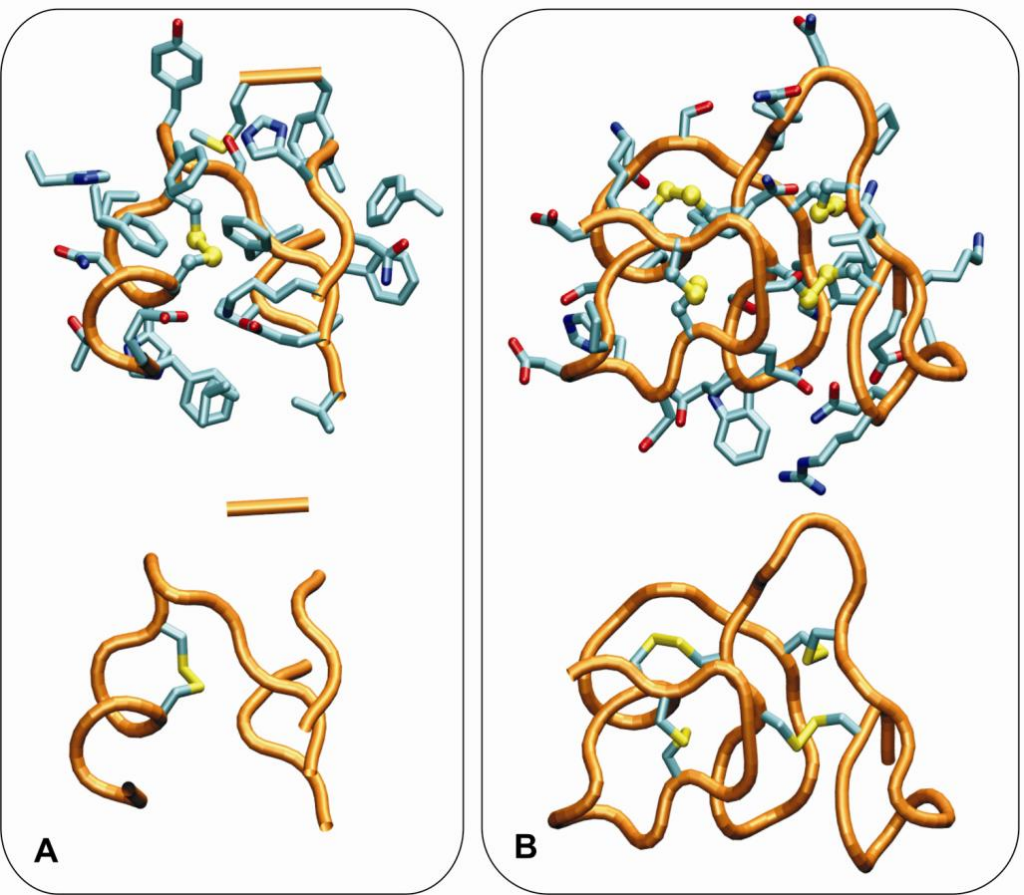
Representative amino acid disulfide environments (top: all side-chains; bottom: only the side-chains of the cysteines involved in disulfide-bonds are depicted). (**A**) thioredoxin-like (PDB id 1bed); (**B**) *SDP*’s superfamilies (plant defensin, PDB id 1q9b). A cutoff 10 Å around the disulfide bonds was considered.

The known differences between the thioredoxin-like superfamily and the 11 superfamilies with a disulfide-rich fold domain from small proteins class, are confirmed by the values the Scheffé descriptors. These differences include:

(v) Unlike for the thioredoxin-like superfamily, the folding of small disulfide-rich proteins is dependent on disulfide bond cooperative effects—this is evident from the significantly larger relative frequency of cysteine residues observed in the small disulfide-rich proteins (Figure 1A and Figure 4);
(vi) thioredoxin-like proteins have a large hydrophobic core, absent in the small disulfide-rich proteins—this leads to significantly lower frequencies of amino acids from classes *ALI* and *HB* in the small disulfide-rich proteins relatively to the thioredoxin-like proteins (Figure 1B and 1C).

Our results suggest that the amino acid patterns around disulfide bonds might be used as a tool to cluster proteins in a biologically relevant way. This is an interesting feature of disulfide bonds, that to date has never been considered (previous studies [20,21] have only analyzed global statistical tendencies).

## 4. Conclusions

We did a thorough analysis of the amino acid neighborhood of the disulfide bonds using stratified statistics, which implies grouping the various proteins into superfamilies before sampling. We examined both the abundance and the diversity of individual amino acids and amino acid groups.

We found that the regions around disulfide bonds are particularly rich in weakly hydrophilic and aromatic amino acids. Aliphatic and hydrophobic amino acids exhibited the lowest relative abundance.

The diversity, associated with the distribution of the different entities over the sample, was determined by using the *F* descriptor within the *ANOVA* statistics. The results obtained show that the entities with large diversity are those presenting the largest discriminate behavior between the thioredoxin-like and the *SDP* superfamilies (the cysteine residue and classes *SULFUR*, *NHF* and *HB*).

We also evaluated the diversity within each superfamily using the Scheffé distances, which were introduced in this work. A most frequent motif was identified in the protein set. The 3D-cartesian projections of the Scheffé distances reflect essentially the deviations of the diverse superfamilies from this motif. In particular, the high divergence between the thioredoxin-like and the *SDP* superfamilies are clearly evident in this representation. These results suggest the possibility of using the composition of the chemical environment around disulfide bonds as a tool in protein classification of very divergent disulfide-rich proteins.

## Supplementary Material



## Figures and Tables

**Table 1.** The amino acid classes assembled using various physicochemical criteria were clustered into two classification groups.

	**Classes**	**Amino Acids**	**Criteria**
**1**	**ALI**	ALA, ILE, GLY, PRO, VAL, LEU	aliphatic side chain
	**AROM**	TYR, PHE, TRP	aromatic side chain (absorbs UV)
	**SULFUR**	CYS, MET	side chain containing a sulfur atom
	**POL**	SER, THR, ASN, GLN	polar side chain
	**CAR**	ASP, GLU, HIS, LYS, ARG	charged side chain
**2**	**HF**	SER, THR, ASN, GLN, ASP, GLU, HIS, LYS, ARG	hydrophilic
	**HB**	ALA, VAL, LEU, ILE, MET, PHE, TRP	hydrophobic
	**NHF**	GLY, CYS, TYR	weakly hydrophilic
	**NHB**	PRO	weakly hydrophobic

**Table 2.** Set of superfamilies under study. The statistical analyses included all the disulfide bonds identified in this protein set. The values in the last three columns were calculated as sums over all the PDB structures of each superfamily (see PDB ids in Table 1 in Supplementary Material).

**SCOP Superfamily**	**SCOP Class**	**SCOP Fold**	**Dominant Secondary Structure**	**Disulfide Bond Propensity^#^**	**Total Number of PDB Structures**	**Total Number of Disulfide Bonds**	**Total Number of Residues**
Crisp	Small proteins	Crisp domain-like	α	5.3%	6	54	1367
Cystine-Knot	Small proteins	Cystine-Knot cykotines	β	3.7%	13	112	3131
Defensin-like	Small proteins	Defensin-like	β	7.4%	15	47	730
EGF-Laminin	Small proteins	Knottins	β	6.4%	27	121	2253
Omega toxins	Small proteins	Knottins	β	8.9%	28	88	992
Plant lectins	Small proteins	Knottins	β	9.9%	8	100	1045
Small snake toxins	Small proteins	Snake toxins-like	β	6.5%	40	209	3279
Scorpion-like toxins	Small proteins	Knottins	β	7.9%	70	247	3303
BBI	Small proteins	Knottins	β	9.6%	5	33	371
BPTI-like	Small proteins	BPTI-like	α + β	5.1%	12	42	814
Kringle-like	Small proteins	Kringle-like	β	3.7%	12	53	1771
Thioredoxin-like	Alpha and beta proteins	Thioredoxin	α/β	0.8%	43	66	10616
Most frequent motif	Small proteins	Knottins	β	[6.7%, 7.3%]*	-	-	-

# Calculated by equation 11;

* Confidence interval, at a 95% level, for the disulfide bonds propensity of *SDP* structures; EGF: Epidermal growth factor; BBI: Bowman Birk Inhibitors; BPTI: basic pancreatic trypsin inhibitor.
