# Supplementary material for: Amino Acid Patterns around Disulfide Bonds
Source: Int J Mol Sci. 2010 Nov 18;11(11):4673–86. doi: 10.3390/ijms11114673 (PMC3000107; doi:10.3390/ijms11114673)
Supplement: Supplementary file 1 [file ijms-11-04673-s001.pdf]

Supplementary Material

Table 1. Protein structures used in the reference set.

**Superfamily**	**PDB IDs**
Crisp	1bgk, 1rc9, 1roo, 1wvr, 1xx5, 2ddb
Cystine-Knot	1agq, 1aoc, 1b8k, 1bet, 1dz7, 1fzv, 1klc, 1tgj, 1wq8, 1wq9, 1www, 2tgi, 2vpf
Defensin-like	1ahl, 1apf, 1atx, 1b8w, 1bds, 1d6b, 1dfn, 1e4r, 1e4t, 1ews, 1kj5, 1kj66, 1sh1, 1tv0, 1ut3
EGF-Laminin	1apo, 1apq, 1b9w, 1cej, 1edm, 1emn, 1epg, 1esl, 1f7m, 1fsb, 1haf, 1i0u, 1ip0, 1jl9, 1k37, 1klo, 1l3y, 1lr7, 1n1i, 1nzi, 1szb, 1toz, 1tpg, 1urk, 2adx, 2j5h, 2tgf
Omega toxins	1c6w, 1cix, 1d1h, 1dl0, 1eit, 1emx, 1g9p, 1i25, 1i26, 1ie6, 1ju8, 1koz, 1kqi, 1la4, 1lmm, 1lmr, 1lup, 1mb6, 1nix, 1niy, 1omb, 1p8b, 1qk6, 1qk7, 1tyk, 1v7f, 1xi7, 1y29
Plant lectins	1ehh, 1mmc, 1p9g, 1q9b, 1uha, 1ulk, 1uln, 9wga
Small snake toxins	1bte, 1ccq, 1cdt, 1cod, 1cre, 1cxn, 1drs, 1erg, 1f94, 1fas, 1ff4, 1g6m, 1je9, 1jgk, 1kba, 1kbs, 1ks6, 1kxi, 1lsi, 1lxh, 1mr6, 1nor, 1ntn, 1ntx, 1onj, 1plo, 1qi9, 1qkd, 1tfs, 1txa, 1vyc, 2abx, 2ccx, 2cdx, 2crt, 2ctx, 2h5f, 2h7z, 2nbt, 5ebx
Scorpion-like toxins	1acw, 1agt, 1ayj, 1b7d, 1bcg, 1big, 1bk8, 1bkt, 1c49, 1c56, 1chl, 1chz, 1cn2, 1djt, 1du9, 1fh3, 1fjn, 1gps, 1gpt, 1hly, 1hp2, 1i2u, 1i6f, 1ica, 1j5j, 1jkz, 1jxc, 1kv0, 1l4v, 1lir, 1lqh, 1lqq, 1m2s, 1mm0, 1mr4, 1mtx, 1myn, 1n4n, 1n8m, 1nra, 1omy, 1ozz, 1pe4, 1pjv, 1pnh, 1pvz, 1px9, 1q2k, 1qky, 1rj, 1scy, 1seg, 1sis, 1sn4, 1snb, 1sxm, 1tsk, 1txm, 1ugl, 1vnb, 1wm7, 1wm8, 2a7t, 2b3c, 2bmt, 2brz, 2crd, 2ktx, 2pta, 2sn3
BBI	1bbi, 1h34, 1mvz, 1pi2, 2fj8
BPTI-like	1aap, 1bf0, 1bik, 1bpi, 1dem, 1dtk, 1dtx, 1irh, 1jc6, 1kth, 1shp, 1tcp
Kringle-like	1bht, 1h8p, 1i71, 1jfn, 1kdu, 1kiv, 1krn, 1l6j, 1pkr, 1tpk, 2fn2, 5hpg
Thioredoxin-like	1aaz, 1bed, 1dby, 1eej, 1ego, 1ep7, 1f9m, 1fb6, 1fg4, 1fov, 1fvk, 1gh2, 1h75, 1j08, 1jfu, 1kng, 1kte, 1o73, 1ovn, 1q98, 1qgv, 1qk8, 1quw, 1qxh, 1r26, 1s3a, 1st9, 1t3b, 1thx, 1v58, 1wou, 1xfl, 1xw9, 1yep, 1zyn, 2a2p, 2a4h, 2b5e, 2b7j, 2cv4, 2cvb, 2ggt, 2ifg

Table 2. *Disulph*'s functionalities.

1	Identification of the disulfide bonds of the sample under study. This sample was considered to include all the disulfide bonds, which occur in the protein set
2	Classification of the twenty coded amino acids in different classes, using three alternative criteria (see Tables 1 and 2)
3	Definition of the neighboring region of a disulfide bond. In the present work, this region was defined as a sphere with a radius of 10 Å centered in the middle point of a disulfide bond
4	Definition spherical shells within the neighboring region. In the present work, twenty shells of this type were considered (see Table 3)
5	For each disulfide bond, identification of the amino acid residues that occur in the respective neighboring region
6	For each disulfide bond, determination of the frequency of each residue in its neighboring region.
7	For each disulfide bond, determination of the frequency of each class in the respective neighboring region
8	For each disulfide bond, determination of the relative frequency of each entity in its neighboring region. For this purpose, two reference sets are considered: the set superfamilies under study presented in Table 4 and a set of proteins selected from PDB database (Xia and Xie, [31])
9	For the sample under study, determination of the density of each residue in the twenty spherical shells defined in 4)

Table 3. Spherical shells within the neighboring region of a disulfide bond, defined as a sphere with a radius of 10 Ǻ centered in the middle point of this bond.

Spherical shell	Distance intervals in Å	Volume/$Å^3$
1	[0.0.0.5]	0.52
2	[0.5.1.0]	3.67
3	[1.0.1.5]	9.95
4	[1.5.2.0]	19.37
5	[2.0.2.5]	31.94
6	[2.5.3.0]	47.65
7	[3.0.3.5]	66.50
8	[3.5.4.0]	88.49
9	[4.0.4.5]	113.62
10	[4.5.5.0]	141.90
11	[5.0.5.5]	173.31
12	[5.5.6.0]	207.87
13	[6.0.6.5]	245.57
14	[6.5.7.0]	286.41
15	[7.0.7.5]	330.39
16	[7.5.8.0]	377.51
17	[8.0.8.5]	427.78
18	[8.5.9.0]	481.19
19	[9.0.9.5]	537.74
20	[9.5.10.0]	597.43

Table 4. Relative frequencies of the amino acids/classes in the sample.

Residue/Class	Relative Frequency	F
CYS	805.60%	122.8
TYR	57.80%	16.7
TRP	45.00%	19.5
ASN	30.50%	34.2
ARG	21.50%	7.3
LYS	16.90%	26
GLY	10.30%	44.7
PRO	5.60%	18.4
SER	−2.50%	14.4
HIS	−3.80%	8.6
THR	−7.40%	28.3
GLN	−13.80%	19.8
PHE	−14.30%	18.6
ASP	−20.00%	14.3
GLU	−25.80%	9.7
ILE	−37.10%	16.3
VAL	−40.90%	26.4
ALA	−46.70%	15.3
LEU	−47.10%	6.6
MET	−56.20%	6.5

Table 4. *Cont.*

SULFUR	228.80%	78.3
NHF	116.40%	72.4
AROM	23.80%	25.2
NHB	5.60%	18.4
POL	1.20%	25.5
HF	−1.30%	12.7
CAR	−3.30%	11
ALI	−28.20%	23
HB	−38.30%	56.2
